# Reduced Levels of Membrane-Bound Alkaline Phosphatase Are Common to Lepidopteran Strains Resistant to Cry Toxins from *Bacillus thuringiensis*

**DOI:** 10.1371/journal.pone.0017606

**Published:** 2011-03-01

**Authors:** Juan Luis Jurat-Fuentes, Lohitash Karumbaiah, Siva Rama Krishna Jakka, Changming Ning, Chenxi Liu, Kongming Wu, Jerreme Jackson, Fred Gould, Carlos Blanco, Maribel Portilla, Omaththage Perera, Michael Adang

**Affiliations:** 1 Department of Entomology and Plant Pathology, University of Tennessee, Knoxville, Tennessee, United States of America; 2 Department of Entomology, University of Georgia, Athens, Georgia, United States of America; 3 State Key Laboratory of Plant Disease and Insect Pests, Institute of Plant Protection, Chinese Academy of Agricultural Science, Beijing, People's Republic of China; 4 Genome Science and Technology Program, University of Tennessee, Knoxville, Tennessee, United States of America; 5 Department of Entomology, North Carolina State University, Raleigh, North Carolina, United States of America; 6 Animal and Plant Health Inspection Service, Biotechnology Regulatory Services, United States Department of Agriculture, Riverdale, Maryland, United States of America; 7 Southern Insect Management Research Unit, Agricultural Research Service, United States Department of Agriculture, Stoneville, Mississippi, United States of America; 8 Department of Biochemistry and Molecular Biology, University of Georgia, Athens, Georgia, United States of America; University of California Merced, United States of America

## Abstract

Development of insect resistance is one of the main concerns with the use of transgenic crops expressing Cry toxins from the bacterium *Bacillus thuringiensis*. Identification of biomarkers would assist in the development of sensitive DNA-based methods to monitor evolution of resistance to Bt toxins in natural populations. We report on the proteomic and genomic detection of reduced levels of midgut membrane-bound alkaline phosphatase (mALP) as a common feature in strains of Cry-resistant *Heliothis virescens*, *Helicoverpa armigera* and *Spodoptera frugiperda* when compared to susceptible larvae. Reduced levels of *H. virescens* mALP protein (HvmALP) were detected by two dimensional differential in-gel electrophoresis (2D-DIGE) analysis in Cry-resistant compared to susceptible larvae, further supported by alkaline phosphatase activity assays and Western blotting. Through quantitative real-time polymerase chain reaction (qRT-PCR) we demonstrate that the reduction in HvmALP protein levels in resistant larvae are the result of reduced transcript amounts. Similar reductions in ALP activity and mALP transcript levels were also detected for a Cry1Ac-resistant strain of *H. armigera* and field-derived strains of *S. frugiperda* resistant to Cry1Fa. Considering the unique resistance and cross-resistance phenotypes of the insect strains used in this work, our data suggest that reduced mALP expression should be targeted for development of effective biomarkers for resistance to Cry toxins in lepidopteran pests.

## Introduction

Cry toxins produced as crystalline inclusions by the bacterium *Bacillus thuringiensis* (Bt) are the most widely used insecticidal trait in transgenic crops for insect control [1]. Due to the wide adoption of Bt transgenic crops, the future efficacy of this technology is threatened by the evolution of resistance by target pests. After more than a decade of commercialization, recent reports support field-evolved resistance to Bt crops in *Helicoverpa zea* [2], *Spodoptera frugiperda* [3], and *Busseola fusca* [4]. Key to the implementation of strategies to delay and manage resistance outbreaks in field environments is the development of efficient methods for early detection. Development of successful DNA-based monitoring methods relies on the identification of biomarker molecules that are specifically and consistently altered in resistant insects. Optimally, resistance biomarkers should efficiently differentiate susceptible and resistant insects, independent of the resistance mechanism, Bt crop, or Cry toxin involved. However, the multi-step mode of Cry toxin action and the diverse resistance mechanisms described to date [5], [6] highlight the difficulty of identifying biomarkers with these ideal characteristics.

Cry toxins target the insect midgut cells to compromise the gut epithelium barrier and facilitate the onset of septicemia [7]. Although the specific mechanism resulting in enterocyte death is still controversial [8], commonly accepted steps in the intoxication process include solubilization of the crystal toxin and activation by the insect gut fluids. Activated toxins are attracted to the brush border membrane of the midgut cells through low affinity binding to glycosylphosphatidylinositol-anchored (GPI-) proteins [9], such as aminopeptidase-N (APN) or membrane-bound alkaline phosphatase (mALP). This initial binding step facilitates subsequent binding of higher affinity to cadherin-like proteins [10], which leads to further processing of the toxin, resulting in formation of a toxin oligomer. Toxin oligomers display high binding affinity towards N-acetylgalactosamine (GalNAc) residues on GPI-anchored proteins [11], resulting in concentration of toxin oligomers on specific membrane regions called lipid rafts, where they insert into the membrane forming a pore that leads to osmotic cell death [12]. Alternatively, binding of toxin monomers to cadherin has been reported to activate intracellular signaling pathways that result in cell death by oncosis [13].

Based on the crucial role of toxin binding to cadherin and the observation that mutations in cadherin genes are linked with resistance to Cry1Ac in *Heliothis virescens* [14], *Helicoverpa armigera* [15], and *Pectinophora gossypiella* [16], DNA-based assays to detect cadherin-gene alterations have been tested for resistance detection [17], [18]. However, the existence of alternative resistance mechanisms [19], [20], [21], [22] suggests that, at least in some cases, tests based on detection of cadherin alterations would be inefficient in detecting Bt resistance.

The main goal of the present study was to identify an efficient biomarker for resistance to diverse Cry toxins. Using differential proteomics (2D-DIGE), we detected reduced levels of mALP from *H. virescens* larvae (HvmALP) in three strains displaying diverse Cry resistance phenotypes when compared to susceptible larvae. Quantitative RT-PCR data supported that this reduction in HvmALP levels was due to reduction in amounts of HvmALP transcripts. Reduced levels of HvmALP homologues were also detected for a Cry1Ac-resistant strain of *H. armigera*, and Cry1Fa-resistant strains of *S. frugiperda*, further evidence supporting the potential development of resistance monitoring methods using reduced mALP levels as an efficient Cry resistance biomarker in lepidopteran insects.

## Materials and Methods

### Insect strains

*H. virescens* laboratory strains YDK, YHD2-B, CXC, and KCBhyb have been previously described [21], [23], [24]. Briefly, YDK is an un-selected susceptible strain, while YHD2-B was generated after continuous selection of larvae from the YHD2 strain with Cry1Ac. Both CXC and KCBhyb originated by selecting with Cry2Aa the offspring from backcrosses of moths from Cry1Ac/Cry2Aa resistant strains (CP73-3 and KCB, respectively) to susceptible adults. Both CXC and KCBhyb larvae were resistant to Cry1Ac (200- to 300-fold) and Cry2Aa (more than 250-fold) when compared to YDK larvae [22]. In contrast, YHD2-B larvae are 73,000-fold resistant to Cry1Ac [25], but display only low levels (4 to 25-fold) of cross-resistance to Cry2Aa [23].

The Cry1Ac-susceptible strain of *H. armigera* 96S was originally collected from Xinxiang County (Henan Province, P. R. China) in 1996, and the larvae have since been reared in the laboratory on an artificial diet without exposure to any Bt toxins or chemical insecticides. The *H. armigera* Cry1Ac-resistant strain BtR was derived from 96S by selection with Cry1Ac protoxin incorporated into the diet [26]. After continuous selection for 87 generations, larvae of this strain display more than 2,900-fold resistance to Cry1Ac when compared to 96S larvae.

The 456 and 512 strains of *S. frugiperda* were originated from isofamilies of insects collected in Puerto Rico in 2009 [27]. Two Cry-susceptible strains of *S. frugiperda* were used as reference in our studies. Eggs of one of the strains (Mon) were kindly supplied by Nancy Adams (Monsanto), while eggs of the second strain (Ben) were purchased from Benzon Research (Carlisle, PA).

All insects were reared in the laboratory using artificial diet as previously described [23], [26]. Fifth instar larvae from each strain were dissected, and midguts frozen and kept at −80°C until used to prepare BBMV as described below, or placed in RNA*later* (Ambion) overnight at 4°C and then stored at −80°C.

### BBMV purification

BBMV were isolated by the differential centrifugation method of Wolfersberger *et al.* [28] with minor modifications for *H. virescens* and *S. frugiperda* [25]. BBMV proteins were quantified by the method of Bradford [29], using BSA as standard, and kept at −80°C until used. Specific activity of N-aminopeptidase (APN) using leucine-*p*-nitroanilide as substrate was used as a marker for brush border enzyme enrichment in the BBMV preparations. APN activities in the final BBMV preparations from all insect species were enriched 5–8 fold when compared to initial midgut homogenates.

### 2D Differential In Gel electrophoresis (2D-DIGE) analysis of BBMV proteomes

BBMV proteins to be used in 2DE were extracted and precipitated using the 2D Clean-Up Kit (GE Healthcare). Precipitated proteins were dissolved in solubilization buffer (5 M urea [Plus-One; GE Healthcare], 2 M thiourea [Sigma], 2% CHAPS [Plus-One, GE Healthcare] and Complete™ protease inhibitors cocktail [Roche]). After centrifugation at 15,700× *g* for 10 min, solubilized proteins in the supernatant were quantified using the 2D Quant Kit (GE Healthcare) following manufacturer's instructions. BBMV proteins (50 µg per sample) were minimally labeled with Cy3 or Cy5 CyDyes (GE Healthcare) following manufacturer's instructions. Three replicates for each strain from independent BBMV preparations were used. Additionally, samples were also reverse CyDye labeled to account for possible differential labeling effects.

BBMV protein samples (50 µg) were used to rehydrate 11 cm (for Western blots) or 18-cm (DIGE analysis) and pH range 4–7 Immobiline DryStrips (GE Healthcare) overnight in rehydration buffer (solubilization buffer plus 0.002% bromophenol blue, 0.018 M dithiothreitol [DTT], and 0.5% ampholytes). Solutions used to rehydrate 18 cm pH 4–7 Immobiline strips for the DIGE analysis contained three samples labeled with a distinct CyDye (Cy2, Cy3, and Cy5). The Cy3and Cy5 labeling was used for experimental samples (three biological replicates for each strain, each sample was labeled with Cy3 and Cy5 to confirm lack of labeling bias), while the Cy2-labeled sample consisted of equal amounts of all the analyzed BBMV protein samples (50 µg total) and was used as internal reference for comparison of diverse gels. Following rehydration, strips were subjected to isoelectric focusing using a Multiphor II unit following manufacturer's recommendations (GE Healthcare). Temperature was maintained at 20°C throughout focusing. Focused strips were equilibrated for 15 min in equilibration buffer (6 M urea [Plus-One; GE Healthcare], 2% SDS, 30% glycerol, 0.05 M Tris [pH 8.8], 0.002% bromophenol blue) containing 1% DTT followed by a second equilibration for 15 minutes in equilibration buffer plus 4% iodoacetamide. For second dimension separation we used the Ettan Dalt*six* system (GE Healthcare) and SDS-8% PAGE gels following manufacturer's instructions. Separated proteins were fixed in 30% ethanol, 7.5% acetic acid overnight at room temperature for DIGE analysis.

Gels were imaged with a Typhoon 9400 scanner (GE Heatlhcare), optimizing the photomultiplier tubes for each laser to achieve the broadest dynamic range. Wavelengths for the filters/lasers were 532 nm/580 nm for Cy3 and 633 nm/670 nm for Cy5. Gel images were analyzed using DeCyder software (GE Healthcare). Reference maps were obtained for each gel using the Cy2-labeled sample and spot correspondence established to compare protein spot abundance within and between gels. Two-fold differences in spot volume were considered as relevant between samples. Statistical significance was estimated using one-way Analysis Of Variance (ANOVA) in the DeCyder software.

Protein identification using peptide mass fingerprinting (PMF) was done at the University of Georgia Proteomics Facility as described elsewhere [30]. Generated PMF data were used in correlative searching strategies to search the Metazoan subset of NCBI using ProFound (http://prowl.rockefeller.edu/) with a confidence level of 0.1 Da and methionine oxidation as a modification.

### Quantification of alkaline phosphatase (ALP) and aminopeptidase (APN) activities

Specific ALP and APN enzymatic activities of BBMV proteins (1 µg) from *H. virescens*, *H. armigera*, and *S. frugiperda* were measured as described elsewhere [31], except that for *H. armigera* BBMV specific ALP activity was determined using a commercial kit (Alkaline phosphatase, Hou-Bio, P. R. China). Enzymatic activities were monitored for 2–5 min. as changes in OD at 405 nm wavelength at room temperature in a microplate reader (BioTek), and the maximum initial velocity (Vmax) calculated using the associated KC4 Data Analysis Software. One enzymatic unit was defined as the amount of enzyme that would hydrolyze 1.0 µmole of substrate to chromogenic product per minute at the specific reaction pH and temperature. Data shown are the mean specific activities from at least three independent BBMV batches from each strain measured in at least three independent experiments. Statistical significance was determined through analysis of variance (ANOVA) using Holm-Sidak or Tukey's multiple pairwise comparison tests (overall significance level = 0.05), using SigmaPlot v.11.0 software (Systat Software Inc., San Jose, CA, USA). Since APN activity data failed an equal variance test, in this case we used ANOVA on ranks (Kruskal-Wallis test, overall significance level = 0.05) to determine statistical significance.

### Western blotting

BBMV proteins to be analyzed by one-dimension electrophoresis (1D) were solubilized in sample buffer [32]. Solubilized BBMV proteins (20 µg) were then heat-denatured for 5 min. and loaded on SDS-8% PAGE gels. Following electrophoretic separation BBMV proteins were transferred overnight at 4°C to polyvinylidene difluoride Q (PVDF) membrane filters (Millipore) at 20 V constant voltage. Filters were blocked for one hour in PBST (135 mM NaCl, 2 mM KCl, 10 mM Na_2_HPO_4_, 1.7 mM KH_2_PO_4_, pH 7.5, 0.1% Tween-20) plus 3% BSA. After blocking, all filter incubations and washes were done in PBST plus 0.1% BSA. Blocked filters were probed with antisera against *Bombyx mori* mALP (a gift from Dr. Masanobu Itoh, Kyoto Institute of Technology, Japan), *Anopheles gambiae* mALP (a gift from Dr. Gang Hua, University of Georgia, USA), *M. sexta* 120-kDa APN [33], or HevCaLP cadherin [21], to detect HvALP, *S. frugiperda* mALP, APN and cadherin on BBMV. Goat anti-rabbit antibodies conjugated with horseradish peroxidase (HRP) were used as secondary antibodies and blots developed using enhanced chemiluminescence (West pico, Pierce).

### Quantitative real-time PCR

Total RNA was extracted from frozen midguts using an RNeasy mini kit (Qiagen) for *H. virescens* or Trizol reagent (Invitrogen) for *H. armigera* samples. Purified RNA was subjected to DNaseI treatment (Takara) to remove any residual DNA according to the manufacturer's instructions. For *H. virescens* samples, total RNA was quantified using RiboGreen® reagent (Molecular Probes) and a fluorescence microplate reader (FLUOstar GALAXY; BMG). The integrity of total RNA was verified by the visualization of a distinct band corresponding to 18S rRNA resolved on a 1.2% formaldehyde agarose gel. First strand cDNA was synthesized in reactions containing 2 µg of total RNA pooled from 5 *H. virescens* midguts using PowerScript™ reverse transcriptase (Clontech), anchored oligo(dT)_20_ primer (Invitrogen) and other reaction components in a 10 µl reaction. For samples from *H. armigera* larvae, total RNA was reverse-transcribed with SuperScript III RNaseH^−^ reverse transcriptase (Invitrogen).

The first strand cDNA was used as a template for qRT-PCR. For *H. virescens* samples, we designed specific primers to amplify 100-bp fragments of an internal region in HvmALP1 (accession no. FJ416470) and HvmALP2 (accession no. FJ416471) isoforms of HvmALP. These HvmALP isoforms were selected because they displayed the highest sequence divergence [34] and the targeted region could be used to differentiate between these two HvmALP isoforms. Forward primer 5′ GAT TTA GGA CGC GAC AGT ATG 3′ and reverse primer 5′ CAG CGG TAA CAT CTG GTC GAA 3′ were used to amplify HvmALP1, while forward primer 5′ GGG ATG TTG ATC TAG ACA ACG T 3′ and reverse primer 5′ CAG CTG TAA CAT CTG GTC GAA T 3′ were used to amplify HvmALP2. As endogenous control, we amplified ribosomal protein S18 (RpS18) using forward 5′ ATG GCA AAC GCA AGG TTA TGT TT 3′ and reverse 5′ TTG TCA AGA TCA ATA TCG GCT TT 3′ primers designed based on the *B. mori* RpS18 sequence (accession no. AY69334).

For *H. armigera* samples, primers were made to amplify a 128 bp conserved region between the HaALP1 (accession no. EU729322) and HaALP2 (accession no. EU729323) isoforms. TaqMan probes (Invitrogen) were labeled at the 5′ end by the reporter dye FAM and at the 3′end by the quencher dye TAMRA. Forward primer 5′ ATA GGC GTA GAC GGC ACG G 3′, reverse primer 5′ CGA GTC GTC GTC ACA ATA CCG 3′, and 5′-FAM CGC CGA GGA GAC TGT CAA GCC GCT T3′-TAMARA were used for HaALP fragment amplification. As endogenous control, we amplified a 184 bp fragment of *H. armigera* actin (accession no. X97615) with forward primer 5′ CAC AGA TCA TGT TCG AGA CGT TCA A 3′, reverse primer 5′- GCC AAG TCC AGA CGC AGG AT-3′ and 5′-FAM CCG CCA TGT ACG TCG CCA TCC AGG 3′-TAMARA.

Quantitative RT-PCR reactions were conducted with three technical replicates for each of three independent biological samples on an ABI 7900HT fast real-time PCR system (Applied Biosystems). PCRs for each template and primer combination were conducted in triplicate and replicated with cDNA prepared from at least three independent biological samples. For *H. virescens* samples reactions (12.5 µl) consisted of a cDNA equivalent of 20 ng of total RNA, 6.25 µl Power® SYBR Green PCR Master Mix (Applied Biosystems) and forward and reverse primers at 0.9 µM concentration. Reactions for *H. armigera* samples (25 µl) consisted of 12.5 µl of *Premix Ex* Taq (2×) (TaKaRa), 0.5 µl of Rox Reference Dye (50×), probe (0.2 µM), primers (0.4 µM), 1 µl of sample cDNA and sterilized ultrapure H_2_O (Millipore). Amplification conditions consisted of an initial denaturation at 95°C for 10 min followed by 40 cycles of 95°C for 15 s, 58°C for 30 s and 72°C for 30 s for *H. virescens* samples, while for *H. armigera* samples a single step for annealing and extension was done at 60°C for 60 s. The absolute value of the slope (Ct value Vs Log) for each primer set was <0.1 and all amplification efficiencies when compared to the endogenous control were >99%, indicating a passing validation. Data obtained were analyzed with the relative 2^−ΔΔCT^ quantitation method to calculate transcript abundance [35], using S18 (*H. virescens*) or actin (*H. armigera*) as internal standards. Transcript amounts were standardized to 1 with the sample from susceptible larvae containing the highest transcript levels from the three biological replicate reactions performed. Upon completion of a quantitative PCR run, a dissociation curve analysis was conducted to verify the absence of any nonspecific amplicons. Statistical significance was tested with ANOVA in the SigmaPlot v.11.0 software, using the Holm-Sidak method (overall significance level = 0.05) for multiple comparisons.

## Results

### Reduced levels of alkaline phosphatase are common to diverse Cry-resistant *H. virescens* strains

**Figure 1:**
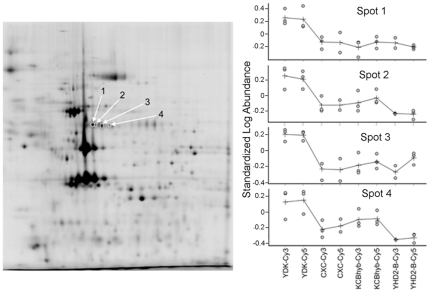
Detection of reduced HvmALP expression in Cry-resistant strains of *H. virescens* using quantitative differential proteomic analysis (2D-DIGE). BBMV proteins from susceptible (YDK) and resistant (YHD2-B, CXC, and KCBhyb) larvae were fluorescently labeled and the corresponding sub-proteome analyzed using 2D-DIGE. A representative gel image is presented with arrows pointing to the four HvmALP spots detected with lower expression levels. The identity of these spots as HvmALP was obtained by peptide mass fingerprinting, de novo sequencing, and Western blotting with specific antisera (data not shown). The standardized log abundance for each spot in all three BBMV samples (labeled with Cy3 or Cy5) from each strain used is shown. Differences in protein levels between HvmALP spots in BBMV from susceptible and resistant larvae were statistically significant (p<0.001; Student T-test). HvmALP expression levels among BBMV samples from resistant larvae were not significantly different.

In an attempt to identify resistance biomarkers, we used a differential proteomics approach (2D-DIGE) to compare BBMV proteomes from susceptible (YDK) and three resistant *H. virescens* strains (CXC, KCBHyb, and YHD2-B) displaying diverse resistance phenotypes [36]. In these assays we detected four protein spots (Fig. 1) that were significantly down-regulated (3 to 4-fold) in BBMV from all resistant larvae compared to vesicles from susceptible insects (one-way ANOVA, P<0.05). In contrast, no significant differences in the levels of these four protein spots were found among BBMV from the resistant strains. All four protein spots were previously identified as membrane-bound alkaline phosphatase using mass spectrometry and Western blotting [30], [37].

**Figure 2:**
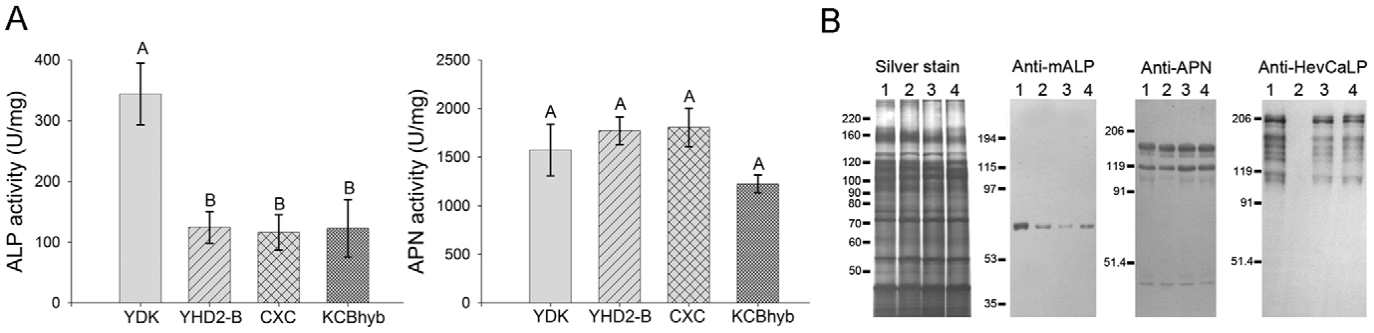
BBMV from Cry1Ac-resistant *H. virescens* larvae display reduced ALP levels. (A) BBMV proteins from *H. virescens* strains as indicated were used in specific ALP or APN activity assays. Different letters indicate statistically significant differences (p<0.05; Holm-Sidak method) among the samples. (B) Western blot analysis of HvmALP, APN, or HevCaLP in BBMV proteins from *H. virescens* strains: Lane 1, YDK; lane 2, YHD2-B; lane 3, CXC; lane 4, KCBhyb.

To further examine the reduction of alkaline phosphatase in BBMV from resistant compared to susceptible insects, we performed ALP activity assays. Compared to YDK samples, specific ALP activity in all resistant strains was significatively reduced (P<0.05, Holm-Sidak method) by about 50% (Fig. 2A). In contrast, activity of another brush border membrane enzyme, APN, was not statistically different among the BBMV samples (P = 0.415, Kruskal-Wallis ANOVA on ranks). In agreement with these data, antisera against membrane-bound alkaline phosphatase from *B. mori* detected lower levels of HvmALP protein in all resistant strains compared to YDK samples, while the pattern of BBMV proteins reacting to antisera against APN appeared unchanged among strains, and cadherin (HevCaLP) expression was only reduced in BBMV from YHD2-B larvae (Fig. 2B).

### Reduced expression of HvmALP is due to reduced transcript levels

**Figure 3:**
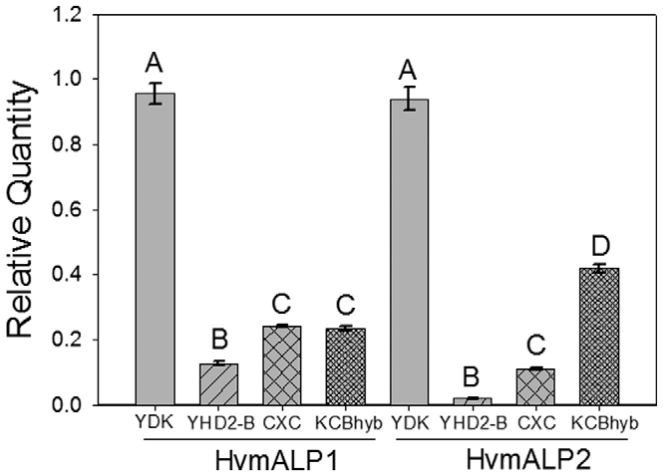
Reduced levels of HvmALP1 and HvmALP2 transcripts in Cry1Ac-resistant *H. virescens* larvae relative to YDK as detected by qRT-PCR. Data shown are the mean transcript quantity relative to the YDK sample with the highest transcript amounts from three independent biological replicates for each HvmALP isoform and strain. All reactions were performed with triplicate technical replicates. Bars denote standard error of the mean; different letters on each bar indicate significant differences (P<0.05; Holm-Sidak method).

To further investigate the mechanism resulting in reduced HvALP expression levels in BBMV from resistant *H. virescens* larvae, we used qRT-PCR to detect HvmALP transcript levels in midguts from susceptible and resistant *H. virescens* larvae. We targeted two HvmALP isoforms displaying the highest degree of sequence divergence (6%) of all the described HvmALP isoforms [34]: HvmALP1 (FJ416470) and HvmALP2 (FJ416471). In our qRT-PCR assays (Fig. 3), both HvmALP1 and HvmALP2 had reduced transcript levels in all resistant when compared to susceptible larvae. The relative levels of transcript reduction were different for each HvmALP isoform. Thus, the biggest reduction in transcript levels was observed for YHD2-B larvae (7.8 fold for HvmALP1 and 59 fold for HvmALP2), while CXC and KCBhyb levels were similar for HvmALP 1 (about 4 fold) but different in the case of HvmALP2 (9 fold for CXC and 2.3 fold for KCBhyb). In all cases, the reduction in levels of HvmALP transcript in resistant larvae was statistically significant (P<0.05, Holm-Sidak method) when compared to the levels detected in samples from susceptible (YDK) larvae.

### Detection of reduced expression of mALP in Cry-resistant *H. armigera* and *S. frugiperda* larvae

**Figure 4:**
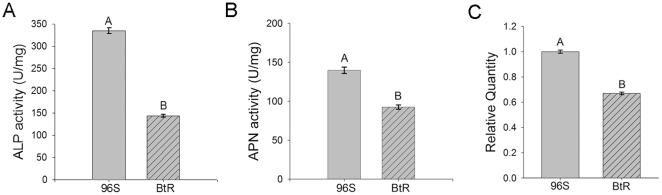
Reduced ALP activity correlates with reduced levels of HaALP transcripts in Cry1Ac-resistant *H. armigera* larvae. BBMV proteins from susceptible (96S) and Cry1Ac-resistant (BtR) *H. armigera* strains were used in specific ALP (A) or APN (B) activity assays. Different letters indicate statistically significant differences (p<0.05; Holm-Sidak method) among the samples. C) Mean relative transcript quantity of HaALP1 and HaALP2 isoforms. Data shown are the mean transcript quantity relative to the 96S sample with the highest transcript amounts from three independent biological replicates for each HaALP isoform and strain. All reactions were performed with triplicate technical replicates. Bars denote standard error of the mean. Different letters indicate significant differences (P<0.05; Holm-Sidak method).

Considering that efficient biomarkers would allow detection of resistance in diverse insects with unique resistance phenotypes, we were interested in testing whether reduced mALP expression levels were common to alternative Cry-resistant lepidopteran species. As a taxonomically-close relative to *H. virescens*, we tested BBMV from susceptible and Cry1Ac-resistant strains of *H. armigera* (Fig. 4A). Similar to tests with *H. virescens* BBMV, we detected a significant reduction (P<0.05, Tukey test) in ALP activity in BBMV from resistant *H. armigera* compared to susceptible larvae. However, in the case of *H. armigera* we also detected significant differences (P<0.05, Tukey test) in APN activity between BBMV from susceptible and resistant larvae (Fig. 4B). Since in *H. virescens* reduced mALP expression was controlled at the transcriptional level, we quantified HaALP transcript amounts in susceptible and Cry1Ac-resistant *H. armigera* larvae using qRT-PCR. In agreement with our HvmALP data, we detected a 1.6-fold reduction (P<0.05, Holm-Sidak method) in levels of HaALP transcripts in guts from resistant larvae compared to susceptible insects (Fig. 4C).

**Figure 5:**
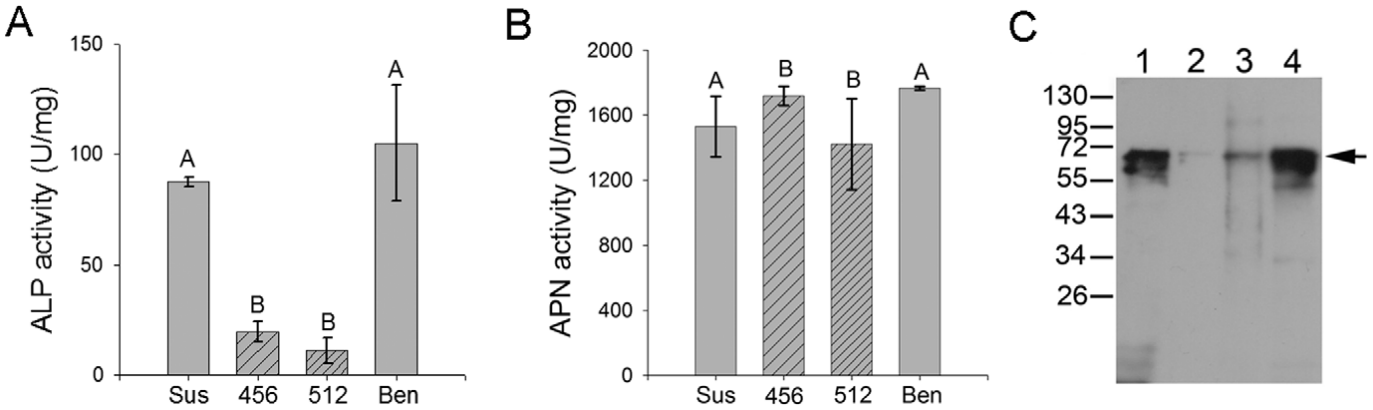
Reduced ALP activity correlates with reduced levels of mALP in BBMV from Cry1Fa-resistant *S. frugiperda* larvae. BBMV proteins from susceptible (Mon and Ben) and Cry1Fa-resistant (456 and 512) strains of *S. frugiperda* were used in specific ALP (A) or APN (B) activity assays. Different letters indicate statistically significant differences (p<0.05; Holm-Sidak method) among the samples. C) Detection of mALP in BBMV from susceptible (Mon and Ben) and resistant (456 and 512) strains of *S. frugiperda* using Western blotting. Lane 1, Mon, lane 2, 456, lane 3, 512, lane 4, Ben. Arrow points to ALP protein band.

To test for reduction of mALP levels in an alternative lepidopteran pest, we used BBMV prepared from susceptible and Cry1Fa-resistant *S. frugiperda* larvae. In activity assays, we detected a significant reduction (P<0.05, Holm-Sidak method) of ALP activity in BBMV from resistant larvae compared to vesicles from susceptible insects (Fig. 5A). Even though we did not detect differences when comparing the two resistant strains (456 and 512) or the two susceptible strains (Mon and Ben), BBMV from resistant larvae presented a 3–4 fold reduction in ALP activity compared to vesicles from susceptible insects. In contrast, we found no significant differences in APN specific activity in BBMV from all four strains (Fig. 5B). Reduction in ALP specific activity correlated with reduced amounts of mALP in BBMV from both resistant strains compared to vesicles from susceptible strains as detected in Western blots (Fig. 5C).

## Discussion

We report on the correlation between reduced ALP protein, activity, and mALP expression levels in strains of three species in the Noctuidae Family with diverse resistance phenotypes against Cry toxins. Currently, *H. virescens* is efficiently controlled by transgenic Bt cotton [38], but numerous reports highlight the potential for the development of field resistance to Bt crops in *H. armigera* [39], [40], [41], while field-evolved resistance to Bt toxins and Bt corn has already been reported for *S. frugiperda* [3], [27]. Considering that the *H. virescens* resistant strains in this study present unique alterations in toxin binding [21], [22], processing of toxin [24], or midgut regeneration [42], [43], our data suggest that reduced mALP expression is a potential biomarker for resistance to diverse Cry toxins and is independent of the resistance mechanism. Furthermore, our data with Cry-resistant Noctuidae suggests that reduced mALP is a common phenomenon in Bt-resistant lepidopteran larvae.

Even though no correlation between ALP activity levels and resistance to insecticides has been reported in the literature to date, reduced ALP activity levels in insects have been reported to occur after intoxication with lectins [44], infection with cytoplasmic polyhedrosis virus (CPV) or *B. thuringiensis* in *B. mori* [45], and microsporidia in *Barathra brassicae* [46]. However, decreased ALP activity appears to be specific to only certain pathologies, since it was not observed after infection of *B. mori* with nuclear polyhedrosis virus (NPV) or *Serratia marcescens* [45]. These observations suggest that although monitoring methods based on reduction in ALP activity levels would allow detection of resistance to Bt toxins, it may need to be combined with additional biomarkers to assure accurate detection of resistance. Another limitation of using reduced ALP levels in monitoring for resistance to Cry toxins is that, considering that in most cases Bt resistance is recessive, heterozygote larvae present similar levels of HvmALP and ALP activity as susceptible parents [31]. To overcome these limitations, we expect that further characterization of the molecular mechanism involved in reduction of ALP levels in Bt-resistant larvae would result in identification of specific alleles to target in designing DNA probes for real time RT-PCR capable of discriminating heterozygotes and ALP reduction due to Bt resistance or infection by entomopathogens.

Previous reports have suggested that direct interaction between *B. thuringiensis* Cry toxins and lepidopteran midgut ALP results in decreased ALP activity [47], [48]. However, our data on the reduction in ALP activity in resistant insects was independent of feeding on Cry-contaminated diet, or on diet composition, as larvae used in our work were reared on diverse diets (including fresh corn leaf tissue) and were not exposed to Cry toxins before dissection. This observation may suggest that a method based on detection of mALP down-regulation would detect Cry-resistant larvae regardless of larvae feeding on transgenic Bt crops or non-Bt refugia.

Considering that mALP has been proposed as receptor for Cry toxins in *M. sexta* [49], *H. virescens* [30], *H. armigera* [50], [51], *Aedes aegypti* [52], *Anopheles gambiae* [53], and *Anthonomus grandis* [54], down-regulation of mALP expression may result in reduced Cry toxin binding to the brush border membrane in resistant insects. However, we detected HvmALP down-regulation in CXC and KCBhyb larvae, while Cry1Ac binding in BBMV from these larvae was not altered when compared to vesicles form susceptible insects [21]. Since Cry1Ac has multiple binding sites in *H. virescens* BBMV [55], and Cry1Ac binding to HvmALP has not been quantified to date, it is possible that changes in Cry1Ac binding due to reduced HvmALP levels are masked by binding to alternative receptors in the BBMV. However, CXC and KCBhyb larvae are cross-resistant to Cry2Aa toxin, which does not share binding sites with Cry1Ac [56].

Mammalian ALP transcript expression is modulated by members of the mitogen-activated protein kinase (MAPK) family. For example, the p38 kinase-dependent pathway modulates changes in ALP activity levels during development or stress in human osteoblast-like [57], [58] and intestinal cells [59]. The homologous p38 pathway in *Caenorhabditis elegans* [60], [61], in *Manduca sexta*, and *A. aegypti* [62] controls the gut defense response against Cry intoxication. Considering this information, it is possible that reduced ALP levels in Cry-resistant insects reflect a constitutively expressed enhanced defensive response to intoxication, as previously suggested for *Ephestia kuehniella* [63] or some of the *H. virescens* resistant strains tested in our work [42], [43]. Based on reported functions for insect ALP enzymes [64], we expect the gut ALP isoforms down-regulated in the tested Bt-resistant larvae are involved in digestion and gut development. However, down-regulation of these enzymes seems not to affect insect development or survival in the field, as larvae from the 456 and 512 strains of *S. frugiperda* used in this paper were generated from field-collected eggs [27] and do not present affected development when reared on corn leaf tissue compared to susceptible insects (data not shown). Lack of a direct correlation between reduced HvmALP levels and levels of resistance in the tested *H. virescens* strains does not support a direct role for HvmALP in resistance, although multiple resistance mechanisms are present in these insects [21], [22], [65]. Further research is needed to determine whether correlation between reduced levels of mALP expression and resistance represents a direct decrease in functional Cry toxin receptors and/or compensatory alterations in resistant larvae.

Development of insect resistance is one of the most crucial issues related to increased adoption rates for transgenic Bt crops. As with other insect pest groups, monitoring for heliothine resistance to Bt is currently performed using the F1 or F2 screening test [38], [66], which are lengthy and labor intensive. As a monitoring alternative, DNA-based methods would greatly increase sensitivity and speed of detection of resistance alleles, but their development is dependent on the identification of resistance alleles. Cadherins that serve as high affinity Bt toxin receptors have been proposed as optimal targets for the development of DNA-based strategies to detect resistance to Cry toxins in field pest populations [17], [67]. However, cadherin resistance alleles have not been detected in field populations of *P. gossypiella* and *H. virescens* [18], [68], possibly due to the low frequency of Bt resistance alleles in field populations. In the case of *P. gossypiella* screening, extensive parallel bioassay evidence indicated that cadherin alleles linked to resistance were rare or absent in *P. gossypiella* populations screened by PCR [69]. Recent reports suggest that the existence of multiple cadherin resistance alleles in field populations of *H. armigera* may hinder successful screening when using DNA-based methods to detect specific cadherin resistant alleles [70]. More recently, lack of expression of an ABC transporter protein in *H. virescens* has been demonstrated to result in lack of Cry1Ac binding and resistance to this toxin, supporting a potential role for this protein during Cry1Ac intoxication [65]. However, monitoring for alterations in Cry toxin receptors, as in the case of cadherin or ABC transporter-based monitoring methods, would only be effective in detecting resistance to Cry toxins binding to these receptors. The introduction of pyramided Bt crops represents increased levels of selection for resistance mechanisms to diverse Cry toxins that do not share receptors. In addition, other mechanisms not involving cadherin modifications are associated with resistance phenotypes [5]. Based on our data, reduced ALP expression represents a biomarker that would detect resistant insects independently of the resistance mechanism or cross-resistance phenotype. Therefore, a biomarker not based on individual DNA sequence but rather diagnostic of a phenotype associated with resistance to Cry toxins, such as the one identified in this study, would be desirable in the development of monitoring methods. Future work aimed at developing effective and sensitive Bt-resistance monitoring methods will include characterization of the molecular mechanism resulting in ALP down-regulation, testing the range of mALP expression in natural field populations, and optimizing biochemical detection of ALP activity associated with resistance alleles in field populations.
